# Increasing cardiac pyruvate dehydrogenase flux during chronic hypoxia improves acute hypoxic tolerance

**DOI:** 10.1113/JP275357

**Published:** 2018-03-05

**Authors:** Michal K. Handzlik, Dumitru Constantin‐Teodosiu, Paul L. Greenhaff, Mark A. Cole

**Affiliations:** ^1^ School of Life Sciences University of Nottingham Medical School Queen's Medical Centre Nottingham UK; ^2^ MRC/Arthritis Research UK Centre for Musculoskeletal Ageing Research UK

**Keywords:** heart, hypoxia, metabolism, pyruvate dehydrogenase complex

## Abstract

**Key points:**

- The cardiac metabolic reprogramming seen in heart diseases such as myocardial infarction and hypertrophy shares similarities with that seen in chronic hypoxia, but understanding of how the hypoxic heart responds to further hypoxic challenge – hypoxic tolerance – is limited.
- The pyruvate dehydrogenase complex serves to control irreversible decarboxylation of pyruvate within mitochondria, and is a key regulator of substrate metabolism, potentially regulating hypoxic tolerance.
- Acute activation of the pyruvate dehydrogenase complex did not improve cardiac function during acute hypoxia; however, simultaneous activation of the pyruvate dehydrogenase complex during chronic hypoxic exposure improved tolerance to subsequent acute hypoxia.
- Activation of the pyruvate dehydrogenase complex during chronic hypoxia stockpiled cardiac acetylcarnitine, and this was used during acute hypoxia. This maintained cardiac ATP and glycogen, and improved hypoxic tolerance as a result.
- These findings demonstrate that pyruvate dehydrogenase complex activation can improve cardiac function under hypoxia.

**Abstract:**

The pattern of metabolic reprogramming in chronic hypoxia shares similarities with that following myocardial infarction or hypertrophy; however, the response of the chronically hypoxic heart to subsequent acute injury, and the role of metabolism is not well understood. Here, we determined the myocardial tolerance of the chronically hypoxic heart to subsequent acute injury, and hypothesised that activation of a key regulator of myocardial metabolism, the pyruvate dehydrogenase complex (PDC), could improve hypoxic tolerance. Mouse hearts, perfused in Langendorff mode, were exposed to 30 min of hypoxia, and lost 80% of pre‐hypoxic function (*P* = 0.001), with only 51% recovery of pre‐hypoxic function with 30 min of reoxygenation (*P* = 0.046). Activation of the PDC with infusion of 1 mm dichloroacetate (DCA) during hypoxia and reoxygenation did not alter function. Acute hypoxic tolerance was assessed in hearts of mice housed in hypoxia for 3 weeks. Chronic hypoxia reduced cardiac tolerance to subsequent acute hypoxia, with recovery of function 22% of pre‐acute hypoxic levels *vs*. 39% in normoxic control hearts (*P* = 0.012). DCA feeding in chronic hypoxia (*per os*, 70 mg kg^−1^ day^−1^) doubled cardiac acetylcarnitine content, and this fell following acute hypoxia. This acetylcarnitine use maintained cardiac ATP and glycogen content during acute hypoxia, with hypoxic tolerance normalised. In summary, chronic hypoxia renders the heart more susceptible to acute hypoxic injury, which can be improved by activation of the PDC and pooling of acetylcarnitine. This is the first study showing functional improvement of the chronically hypoxic heart with activation of the PDC, and offers therapeutic potential in cardiac disease with a hypoxic component.

## Key points

- The cardiac metabolic reprogramming seen in heart diseases such as myocardial infarction and hypertrophy shares similarities with that seen in chronic hypoxia, but understanding of how the hypoxic heart responds to further hypoxic challenge – hypoxic tolerance – is limited.
- The pyruvate dehydrogenase complex serves to control irreversible decarboxylation of pyruvate within mitochondria, and is a key regulator of substrate metabolism, potentially regulating hypoxic tolerance.
- Acute activation of the pyruvate dehydrogenase complex did not improve cardiac function during acute hypoxia; however, simultaneous activation of the pyruvate dehydrogenase complex during chronic hypoxic exposure improved tolerance to subsequent acute hypoxia.
- Activation of the pyruvate dehydrogenase complex during chronic hypoxia stockpiled cardiac acetylcarnitine, and this was used during acute hypoxia. This maintained cardiac ATP and glycogen, and improved hypoxic tolerance as a result.
- These findings demonstrate that pyruvate dehydrogenase complex activation can improve cardiac function under hypoxia.

## Introduction

Altered cardiac energy metabolism is an integral component of the aetiology and progression of heart failure. An increased reliance on carbohydrate as a source of ATP re‐synthesis and suppressed oxidative metabolism are observed in myocardial hypertrophy and following myocardial infarction (Allard *et al*. 1997; Davila‐Roman *et al*. 2002; Osorio *et al*. 2002; de las Fuentes *et al*. 2003; Heather *et al*. 2006), and these impair the heart's total ATP generating capacity (Neubauer, 2007). Development of myocardial hypoxia is also a characteristic of the contributing factors to heart failure and is observed in myocardial hypertrophy (Sano *et al*. 2007; Karamitsos *et al*. 2013; Mahmod *et al*. 2014). The pattern of metabolic reprogramming seen with sustained hypoxia shares many similarities with that seen in the metabolic remodelling following myocardial infarction, which includes a fall in the rates of fatty acid oxidation, increased glycolysis and depressed mitochondrial ATP generation (Heather *et al*. 2012; Cole *et al*. 2016; Mansor *et al*. 2016).

The link between myocardial hypoxia and the development of heart failure is recognised, but there is limited information on how functionally tolerant the heart is to hypoxia, and studies are conflicting. Recently, the finding that severe, chronic hypoxia improved function following myocardial infarction (Nakada *et al*. 2017) further questions cardiac tolerance to oxygen restriction. Animals, either naturally resident at altitude (Schippers *et al*. 2012) or regularly exposed to hypoxia (Bing *et al*. 1972), show cardiac adaptation with an increased emphasis on glycolysis for ATP supply, and an increased tolerance to hypoxia. However, in naive hearts exposed to chronic hypoxia, the information is conflicting, in that tolerance to global ischaemia following prolonged hypoxia is reported to be either unchanged (Asemu *et al*. 1999), improved (Guo *et al*. 2009) or impaired (Milano *et al*. 2010). Interpretation is further complicated by the use of different chronic hypoxic protocols, in both neonatal and adult animals. Evidence describing tolerance of the chronically hypoxic heart to subsequent acute hypoxia is equally inconsistent (Silverman *et al*. 1997; Milano *et al*. 2002; La Padula & Costa, 2005). Recently, we have developed and characterised a moderate hypoxic protocol (Heather *et al*. 2012; Cole *et al*. 2016; Mansor *et al*. 2016), which involves a period of acclimation similar to that employed by humans in transition to high altitude (Holloway *et al*. 2011). In this model, baseline myocardial function was unaffected, underpinned by extensive metabolic reprogramming, but disruption of this apparent adaptation resulted in cardiac dysfunction (Cole *et al*. 2016; Mansor *et al*. 2016). More detailed information on cardiac responses to hypoxia is therefore needed in order to potentially offer therapies for cardiac disease by identifying intermediate metabolic targets.

Chronic hypoxia results in increased glycolytic flux (Cole *et al*. 2016; Mansor *et al*. 2016), and a fall in mitochondrial respiration (Heather *et al*. 2012). Mitochondrial use of pyruvate, the end‐product of glycolysis, is regulated by the mitochondrial pyruvate dehydrogenase complex (PDC). The PDC catalyses the oxidative decarboxylation of pyruvate to acetyl coenzyme A (acetyl‐CoA) and by doing so serves to regulate mitochondrial carbohydrate use. PDC activation is covalently regulated by a family of pyruvate dehydrogenase kinases (PDK1–4) and a family of pyruvate dehydrogenase phosphatases (PDP1–2) (Bowker‐Kinley *et al*. 1998; Huang *et al*. 1998). However, despite full activation of the PDC complex, flux through the PDC reaction can be inhibited by allosteric regulation (Constantin‐Teodosiu *et al*. 1993). Reduced PDC activity and flux have been reported in infarcted or failing hearts (Seymour & Chatham, 1997; Dodd *et al*. 2014), and acute hypoxia is known to reduce PDC activity in skeletal muscle (Parolin *et al*. 2000) and in mouse embryonic fibroblasts for up to 96 h (Kim *et al*. 2006). Therefore inhibition of PDC activity and flux during prolonged hypoxia could therefore be expected but is currently undetermined.

PDC activity and flux can be acutely increased with the PDK inhibitor dichloroacetate (DCA) (Whitehouse & Randle, 1973; Atherton *et al*. 2011). Acutely, DCA has been shown to improve cardiac function following ventricular fibrillation (Azam *et al*. 2015) and following global ischaemia in healthy and hypertrophied hearts (McVeigh & Lopaschuk, 1990; Randall *et al*. 1998; Wambolt *et al*. 2000; Ussher *et al*. 2012), associated with an increase in glucose oxidation. Whether similar effects are apparent during acute hypoxia, rather than the anoxic conditions of ischaemia is unknown. Furthermore, the impact of chronic PDC activation on cardiac adaptation to hypoxia is also unresolved. However, the impaired PDC activity seen in the diabetic heart (Seymour & Chatham, 1997) can be normalised with chronic DCA treatment (Le Page *et al*. 2015), and improves diastolic function. Therefore, assuming cardiac PDC activity is suppressed with chronic hypoxia, prolonged stimulation of the PDC may be beneficial.

We hypothesised, consistent with the available information from ischaemic studies, that acute hypoxic injury could be improved by pharmacologically increasing PDC activity and flux. In chronic hypoxia, we hypothesised that PDC activity and flux would be suppressed, which in turn would reduce the tolerance of the chronically hypoxic heart to an additional hypoxic challenge. We further hypothesised that increasing PDC activity and flux during prolonged hypoxia would offset the impairment of mitochondrial ATP production and functional impairment associated with chronic hypoxia.

## Methods

### Ethical approval

All animal experiments were approved by the University of Nottingham animal welfare committee, compliant with the UK Home Office Animals (Scientific Procedures) Act, 1986, and amended by European Directive 2010/63/EU. The work conformed to *The Journal of Physiology*’s principles and regulations as described in the checklist of Grundy (2015).

### Animals

Eight‐week‐old outbred male CD1 mice were purchased from a commercial breeder (Harlan, UK). Animals were housed on a 12 h light/dark cycle, fed *ad libitum* and kept under controlled conditions of temperature and humidity.

### Acute hypoxia

Animals were killed with a terminal dose of sodium pentobarbitone (60 mg (kg body weight)^−1^ i.p.) and the heart excised and arrested in ice‐cold Krebs–Henseleit (KH) buffer containing (mm): 118 NaCl, 4.7 KCl, 1.2 MgSO_4_, 1.85 CaCl_2_, 0.5 Na_2_EDTA, 11.0 glucose, 25.0 NaHCO_3_ and 1.2 KH_2_PO_4_. Hearts (*n* = 7) were perfused in Langendorff mode at 80 mmHg constant pressure with 200 mL recirculating KH buffer containing 0.4 mm palmitate pre‐bound to 3% bovine serum albumin. The buffer was continually gassed with a mix of 95% O_2_ and 5% CO_2_, with the temperature maintained at 37°C. Cardiac function was measured continuously using a polyethylene balloon placed within the left ventricular lumen inflated to 4–8 mmHg, determining developed pressure (DP) and heart rate (HR). Rate pressure product (RPP) was calculated from the product of HR and DP. Following 30 min of baseline perfusion, hearts were subjected to 30 min hypoxia by replacing 95% O_2_ with 95% N_2_. This reduced buffer [O_2_] to one‐fifth of normoxic levels. Hearts were then returned to normoxia (reoxygenation) for 30 min. Experiments had three end points, with hearts flash frozen following pre‐hypoxic perfusion (*n* = 7), end‐hypoxia (*n* = 7) and end‐reoxygenation (*n* = 7), and stored at −80°C for subsequent analysis. In a separate group of experiments, 1 mm DCA (Sigma, St Louis, MO, USA) was infused to increase cardiac PDC activity either during hypoxia (DCA Hypox/Reox, *n* = 8) or from the onset of reoxygenation (DCA Reox, *n* = 7).

### Chronic hypoxic exposure

Mice (*n* = 26) were housed for 21 days in a normobaric hypoxic chamber (Medical Engineering Unit, University of Nottingham, UK). Chamber oxygen level (inspired O_2_ fraction, $F_{iO_{2}}$) was regulated via a hypoxic generator (Hypoxico, New York, NY, USA). As described previously, $F_{iO_{2}}$ was reduced from 21% to 11% over 7 days and subsequently maintained at 11% for 14 days (Cole *et al*. 2016). Chamber CO_2_ levels did not exceed 0.1%. A separate group of mice (*n* = 24) were fed DCA *per os* in drinking water throughout hypoxic housing (0.75 g L^−1^ neutralised to pH 7.4 with NaOH) resulting in a dose of ∼70 mg kg^−1^ day^−1^. A further group of normoxic animals (*n* = 25) were housed in the same room. Following removal from the hypoxic chamber, in order to stabilise cardiac function, mice breathed room air for 1 h, prior to being killed and heart perfusion as described above.

### Cardiac metabolic flux

Cardiac glycolytic flux was determined in isolated beating hearts as previously described (Cole *et al*. 2016). Briefly, [5‐^3^H]glucose (Perkin Elmer, Chalfont, Bucks, UK) was added to KH buffer and paired aliquots of recirculating buffer were collected at 5 min intervals during heart perfusion. Conversion of [^3^H]glucose to ^3^H_2_O was determined using Dowex anion separation. Net cardiac lactate efflux was determined following assay of timed buffer collections as previously described (Lundholm *et al*. 1963).

### Pyruvate dehydrogenase activity assay

Five to 10 milligrams of frozen tissue was used to determine PDC activity as previously described (Constantin‐Teodosiu *et al*. 1991b). Briefly, PDC activity in its dephosphorylated active form (PDCa) was assayed in a buffer containing NaF and dichloroacetate (DCA), and was expressed as a rate of acetyl‐CoA formation (μmol min^−1^ (mg wet tissue)^−1^ at 37°C).

### Tissue metabolites

Left ventricular tissue was lyophilised and powdered. Muscle acetylcarnitine was determined using a radioenzymatic method (Cederblad *et al*. 1990). Myocardial ATP, ADP, AMP, lactate, total creatine and glycogen were determined using previously described methods (Harris *et al*. 1974). The total adenine nucleotide (TAN) pool was calculated as a sum of ATP, ADP and AMP. The energy charge of the cell was calculated using the following formula, using the method of Atkinson & Walton (1967):

Energy  charge =[ ATP ]+12[ ADP ][ ATP ]+[ ADP ]+[ AMP ]

### Western blotting

An aliquot of the left ventricle was homogenised in ice‐cold buffer containing 50 mm Tris‐HCl, pH 7.5, 1 mm EDTA, 1 mm EGTA, 1% IGEPAL, 0.1% β‐mercaptoethanol and 10 μL mL^buffer−1^ protease inhibitor cocktail. Tissue lysate was centrifuged at 13,000 *g* for 10 min at 4°C and the supernatant stored at −80°C. Homogenate protein content was determined using a bicinchoninic acid assay (Pierce, UK). Protein samples were run on a 12% Bis‐Tris acrylamide gel for ∼2 h at constant 100 V and transferred to a polyvinylidenedifluoride (PVDF) membrane for 2 h at constant 250 mA in a cooled transfer tank. The membrane was blocked for 1 h and incubated overnight at 4°C with rabbit anti‐PDK1 antibody (Cell Signaling, Leiden, the Netherlands), rabbit anti‐PDK2 antibody (Abgent, San Diego, CA, USA), rabbit anti‐PDK4 (Abgent, San Diego, CA, USA) and rabbit anti‐actin antibody (Sigma‐Aldrich, Gillingham, Dorset, UK). Membranes were then washed and incubated with goat anti‐rabbit HRP‐conjugated secondary antibody (R&D Systems, Abingdon, Oxon, UK). Washed membranes were incubated with enhanced chemiluminesence detection solution (Amersham, Bucks, UK) and exposed to X‐ray film (Kodak, Watford, UK).

### Statistical analysis

Repeated measures ANOVA of mixed design was used to test for differences between groups over time. When significant, Sidak *post hoc* tests were subsequently performed on individual comparisons. Statistical significance was set at *P* ≤ 0.05 for all analyses, with results presented as mean ± standard error of mean.

## Results

### DCA infusion during acute hypoxia and reoxygenation does not affect cardiac function

**Figure 1:**
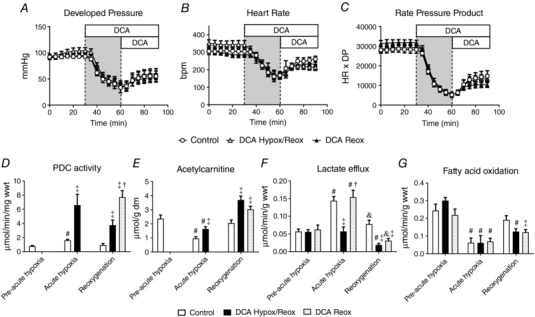
Effect of acute hypoxia (grey bar) and dichloroacetate (DCA) infusion on cardiac function and metabolism (*n* = 7) Developed pressure (*A*), heart rate (*B*), rate pressure product (*C*), cardiac pyruvate dehydrogenase complex (PDC) activity (*D*), cardiac acetylcarnitine content (*E*), lactate efflux (*F*) and fatty acid oxidation (*G*). Hearts were flash frozen at the end of pre‐acute hypoxia, acute hypoxia and reoxygenation for subsequent analysis of metabolites. DCA Hypox/Reox, 1 mm DCA continuously infused from the onset of hypoxia; DCA Reox, 1 mm DCA continuously infused from the onset of reoxygenation. ^#^ *P < *0.05 *vs*. pre‐acute hypoxia in the same group; ^†^ *P* < 0.05 *vs*. DCA Hypox/Reox at a given time point; ^&^ *P* < 0.05 *vs*. acute hypoxia in the same experimental group; ^‡^ *P* < 0.05 *vs*. control at a given time point.

The effects of 30 min of hypoxia on cardiac function, and the influence of DCA infusion during acute hypoxia and reoxygenation were determined compared to control hearts (Fig. 1 *A*–*C*). In control hearts, cardiac function fell steadily throughout acute hypoxia, with a 60% fall in DP (*P* = 0.003) and an 80% fall in RPP after 30 min (*P* = 0.001). Reoxygenation resulted in only partial functional recovery, to 51% of pre‐hypoxic RPP (*P* = 0.046). DCA infusion throughout hypoxia and reoxygenation (DCA Hypox/Reox), or DCA infusion at the onset of reoxygenation (DCA Reox) did not alter end‐hypoxic function, or recovery from hypoxia.

In control hearts, acute hypoxia resulted in a doubling of PDC activity (Fig. 1 *D*, *P* = 0.001), returning to baseline values following reoxygenation. DCA infusion during acute hypoxia increased PDC activity 4‐fold by the end of hypoxia (*P* = 0.008), and with sustained DCA infusion, was still 2‐fold higher by the end of reoxygenation (DCA Hypox/Reox, *P* = 0.002). Infusion of DCA during reoxygenation alone (DCA Reox) increased PDC activity 8‐fold compared to control hearts (*P* = 0.001). In control hearts, acute hypoxia reduced cardiac acetylcarnitine content (Fig. 1 *E*) by 59% at the end of acute hypoxia relative to pre‐acute hypoxia (*P* = 0.001), and acetylcarnitine recovered to pre‐hypoxic levels by the end of reoxygenation. In contrast to the 4‐fold rise in PDC activity with DCA infusion throughout acute hypoxia and reoxygenation, acetylcarnitine only rose by 71% in acute hypoxia compared to control hearts, and was 80% higher by the end of reoxygenation. DCA infusion during reoxygenation alone increased acetylcarnitine concentration by 48% (*P* = 0.017) relative to control hearts at the same time point.

Lactate efflux in control hearts (Fig. 1 *F*) increased 2.5‐fold during acute hypoxia (*P* = 0.001), returning to near the pre‐hypoxic rate with reoxygenation. In contrast, infusion of DCA during hypoxia resulted in lactate efflux being no different from the pre‐hypoxic rate, and the rate at the end of reoxygenation fell compared to control hearts at the same time point (*P* = 0.001). DCA infusion during reoxygenation alone also reduced lactate efflux relative to control hearts at the same time point (*P* = 0.003). Fatty acid oxidation fell by 75% with 30 min of hypoxia (Fig. 1 *G*, *P* = 0.025) and was unaffected by DCA infusion. However, whilst fatty acid oxidation in control hearts recovered to pre‐hypoxic levels with reoxygenation, hearts infused with DCA either throughout hypoxia and reoxygenation, or in the reoxygenation period alone, had 35% lower fatty acid oxidation than control hearts (*P* = 0.047).

### Chronic hypoxia results in reduced tolerance to acute hypoxia

**Figure 2:**
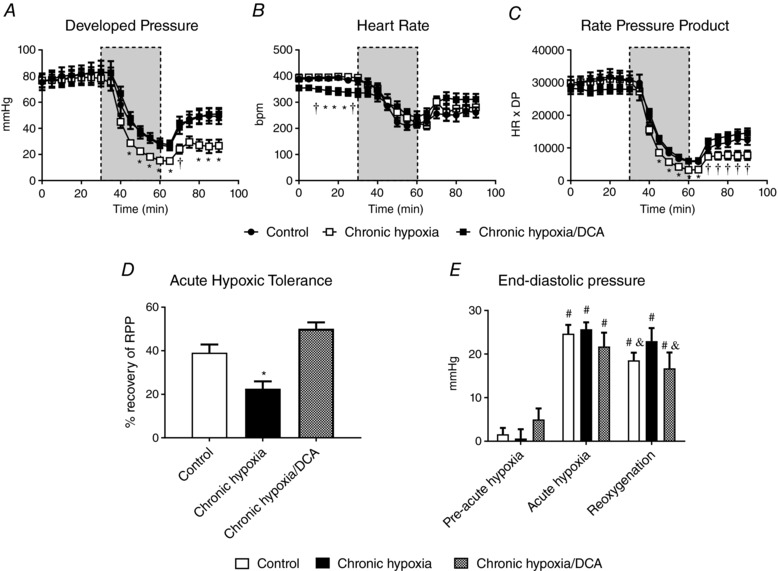
The effect of chronic hypoxia on cardiac function, hypoxic tolerance, and the effect of DCA treatment during hypoxia Developed pressure (DP) (*A*), heart rate (HR) (*B*), rate pressure product (RPP) (*C*), acute hypoxic tolerance (*D*) and end‐diastolic pressure (*E*) following acute hypoxia (grey bar, *n* = 7). ^*^ *P* < 0.05 *vs*. other groups at a given time point; ^†^ *P* < 0.05 *vs*. chronic hypoxia at a given time point; ^#^ *P* < 0.05 *vs*. pre‐acute hypoxia in the same experimental group; ^&^ *P* < 0.05 *vs*. acute hypoxia in the same experimental group.

Mice housed in hypoxic conditions for 3 weeks maintained body weight (Table 1) and had calorific intake no different to normoxic control animals (data not shown). Blood haemoglobin concentration was 71% greater in hypoxic mice (*P* = 0.001). Cardiac function of isolated hearts from chronically hypoxic mice (Fig. 2 *A*–*C*) was no different from normoxic controls, with normal coronary flow rates (Table 1). Acute hypoxia reduced DP of hearts from previously normoxic mice by 64% (Fig. 2 *A*, *P* = 0.001), which was no different to that seen in control hearts in the previous acute DCA study (Fig. 1 *A*), and recovery of function following reoxygenation was also similar to the previous acute study. However, hearts from chronically hypoxic mice were less tolerant of acute hypoxia, end‐hypoxic DP being lower than in hearts from normoxic controls (*P* = 0.014), and final recovery of DP following reoxygenation was 34% of pre‐hypoxic levels, whilst control hearts recovered to 65% (*P* = 0.001). Heart rate was no different between groups during acute hypoxia, and as a result end‐hypoxic RPP was lower in hearts from chronically hypoxic animals and recovery of RPP was impaired relative to normoxic controls (22% *vs*. 39%, *P* = 0.012, Fig. 2 *C* and *D*). End‐diastolic pressure (EDP) had increased 15‐fold by the end of acute hypoxia in hearts from normoxic controls (Fig. 2 *E*, *P* = 0.001), falling slightly with reoxygenation (25%, *P* = 0.007). In contrast, EDP of hearts from chronically hypoxic animals did not fall with reoxygenation, being unaltered relative to normoxic controls at the end of acute hypoxia.

**Table 1:** Animal anthropometric and physiological characteristics

	Body weight (g)	Haemoglobin (g/L)	Heart weight (mg)	HW/BW (mg g^−1^)	CF (mL min^−1^)	CF (mL min^−1^ g^−1^)
Control	39.4 ± 0.7	101.1 ± 5.5	306.6 ± 10.4	7.8 ± 0.3	4.5 ± 0.3	14.9 ± 1.5
Chronic hypoxia	37.8 ± 0.4	173.9 ± 3.4*	305.1 ± 15.7	8.1 ± 0.4	4.9 ± 0.2	16.4 ± 1.4
Hypoxia/DCA	36.4 ± 0.4*	173.0 ± 3.1*	315.0 ± 11.9	9.0 ± 0.3	4.5 ± 0.3	14.3 ± 0.8

Values are means ± SEM (*n* = 8). HW/BW, heart weight/body weight ratio; CF, coronary flow. ^*^ *P* < 0.05 *vs*. control.

### DCA improves hypoxic tolerance of the chronically hypoxic heart

Body weight of chronically hypoxic animals was 8% lower in the DCA‐fed group relative to normoxic controls (*P* = 0.002), but was unaltered relative to chronically hypoxic animals fed with vehicle (Table 1). Blood haemoglobin concentration, heart mass and coronary flow rate were not altered with DCA feeding. Hearts from chronically hypoxic animals fed DCA had normal DP, slightly lower heart rate (*P* = 0.024), but normal RPP (Fig. 2 *A*–*C*) prior to acute hypoxia. DCA protected hearts from chronically hypoxic mice, as the end‐hypoxic DP was greater in hearts from DCA‐treated chronically hypoxic mice than from vehicle‐fed animals (28.7 ± 3.5 *vs*. 15.3 ± 1.4 mmHg, *P* = 0.01), and RPP was also increased (5900 ± 600 *vs*. 3300 ± 300 mmHg (beats min^−1^), *P* = 0.003). DCA increased final recovery of RPP relative to vehicle‐fed chronically hypoxic mice, from 23 ± 6 to 50 ± 3% (Fig. 2 *C* and *D*, *P* = 0.001). This was largely as a consequence of increased recovery of DP (Fig. 2 *A*, *P* = 0.016). In contrast to the maintained EDP seen with reoxygenation in vehicle‐fed chronically hypoxic hearts, DCA resulted in reoxygenation EDP levels being no different to those seen in normoxic controls (Fig. 2 *E*). In summary, DCA feeding during chronic hypoxia improved cardiac function both during an acute hypoxic challenge and in reoxygenation.

### Chronic DCA treatment normalises cardiac glycolytic flux and lactate efflux during reoxygenation

**Figure 3:**
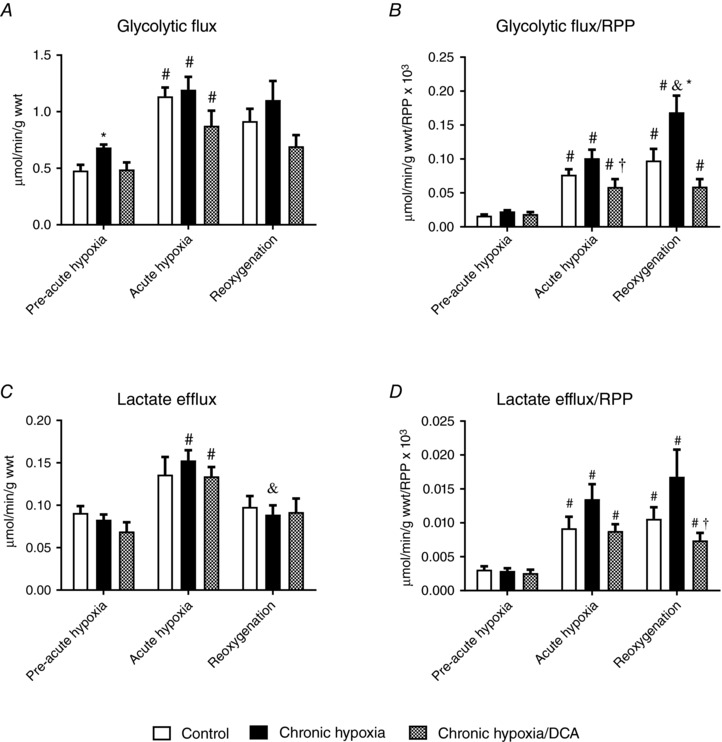
The effect of acute hypoxia and reoxygenation following chronic hypoxia ± DCA on cardiac glycolysis and lactate efflux (*n* = 8) Glycolytic flux (*A*) and normalised to function (*B*). Lactate efflux (*C*) and normalised to function (*D*). ^*^ *P* < 0.05 *vs*. other experimental groups at a given time point; ^#^ *P* < 0.05 *vs*. pre‐acute hypoxia in the same experimental group; ^&^ *P* < 0.05 *vs*. acute hypoxia in the same experimental group; ^†^ *P* < 0.05 *vs*. chronic hypoxia at a given time point.

Chronic hypoxic housing increased cardiac glycolytic flux by 41% relative to normoxic controls (Fig. 3 *A*, *P* = 0.024), but this was normalised by DCA. As expected, the glycolytic rate increased 2.4‐fold during acute hypoxia (*P* = 0.001) in normoxic control hearts, and the rate at the end of acute hypoxia was no different in chronic hypoxic animals irrespective of treatment with DCA. However, when the glycolytic rate was expressed relative to RPP (which was altered with chronic hypoxia – Fig. 2 *C*), DCA‐treated hearts had a 42% lower glycolytic rate at the end of acute hypoxia (*P* = 0.035). During reoxygenation, chronically hypoxic hearts had 1.7‐ and 2.9‐fold greater glycolytic rate relative to both normoxic controls (*P* = 0.038) and DCA‐treated chronically hypoxic hearts (*P* = 0.001), respectively.

Cardiac lactate efflux was not altered with chronic hypoxia with or without DCA (Fig. 3 *C*). Acute hypoxia resulted in a doubling of lactate efflux in hearts from chronically hypoxic animals, which was not affected by DCA (*P* = 0.001). Lactate efflux normalised to cardiac function was not different prior to acute hypoxia (Fig. 3 *D*), but acute hypoxia increased lactate efflux/RPP 1.5‐fold (*P* = 0.022), with similar increases in hearts from chronically hypoxic animals. This was maintained during reoxygenation, but lactate efflux/RRP was 54% lower in hearts from DCA‐fed animals exposed to chronic hypoxia compared to vehicle‐fed animals (*P* = 0.007).

### Chronic DCA treatment leads to acetyl group accumulation

**Figure 4:**
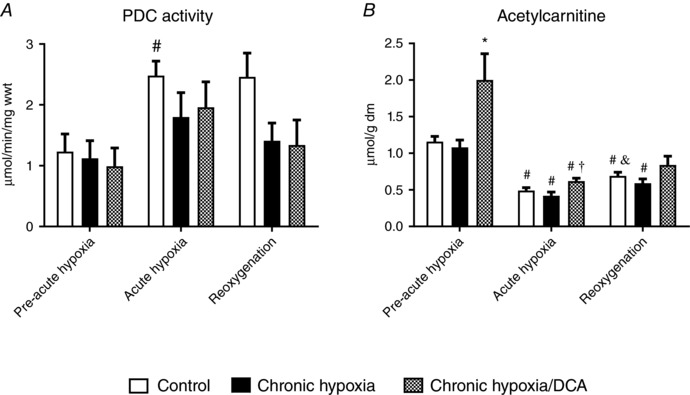
The effect of acute hypoxia and reoxygenation following chronic hypoxia ± DCA on pyruvate dehydrogenase complex (PDC) activity (*A*) and acetylcarnitine (*B*) ^#^ *P* < 0.05 *vs*. pre‐acute hypoxia in the same group; ^*^ *P* < 0.05 *vs*. other groups at a given time point; ^&^ *P* < 0.05 *vs*. acute hypoxia in the same group; ^†^ *P* < 0.05 *vs*. chronic hypoxia at a given time point, *n* = 7–10.

Cardiac PDC activity prior to acute hypoxia was unaltered with chronic hypoxia with or without DCA (Fig. 4 *A*), and levels of PDK1 and PDK4 protein were also unaltered across groups (data not shown). Acute hypoxia doubled PDC activity in hearts from normoxic animals (*P* = 0.032), but not in chronically hypoxic animals or in animals DCA‐treated in chronic hypoxia. Chronically hypoxic animals treated with DCA had double the cardiac acetylcarnitine content prior to acute hypoxia compared to vehicle‐fed animals (Fig. 4 *B*, *P* = 0.013). Consistent with our findings from our first acute hypoxic study (Fig. 1 *E*), acetylcarnitine content fell by the end of acute hypoxia in all groups, and the high level of acetylcarnitine with DCA treatment had returned to near‐control levels (*P* = 0.006). Acetylcarnitine content remained low during reoxygenation under all experimental conditions.

### Chronic DCA treatment preserves cardiac ATP and glycogen content during acute hypoxia

**Figure 5:**
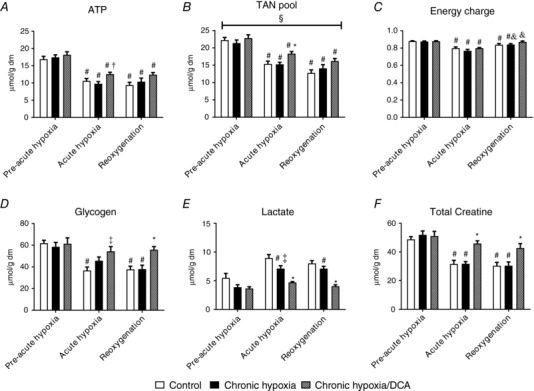
The effect of acute hypoxia and reoxygenation following chronic hypoxia ± DCA on cardiac ATP (*A*), total adenine nucleotide (TAN) pool (*B*), cell energy charge (*C*), glycogen (*D*), lactate (*E*) and total creatine (*F*) ^#^ *P* < 0.05 *vs*. pre‐acute hypoxia in the same group; ^†^ *P* < 0.05 *vs*. chronic hypoxia at a given time point; ^*^ *P* < 0.05 *vs*. other groups at a given time point; ^‡^ *P* < 0.05 *vs*. control at the same time point; ^&^ *P* < 0.05 *vs*. acute hypoxia in the same experimental group; ^§^ *P* = 0.034 main group effect Hypoxia/DCA *vs*. control and chronic hypoxia, *n* = 7–10.

Chronic hypoxia did not alter baseline cardiac ATP content (Fig. 5 *A*), and this was not affected by DCA. Acute hypoxia reduced cardiac ATP by 37% in normoxic control hearts (*P* = 0.001) and by 44% in hearts from chronically hypoxic mice (*P* = 0.001), but chronic DCA feeding preserved 28% more ATP compared to hearts from chronically hypoxic animals (*P* = 0.018). Cardiac ATP was not altered within any group with reoxygenation. Consistent with these observations, the cardiac TAN pool (Fig. 5 *B*) was greater in DCA‐treated animals, both at end‐hypoxia and following reoxygenation (main effect, *P* = 0.034). The energy charge of the cell was unaltered with either chronic hypoxia or DCA treatment (Fig. 5 *C*). Acute hypoxia resulted in a fall in energy charge in control hearts (9%, *P* = 0.006) and was unaltered with chronic hypoxia or DCA treatment. Energy charge remained low in control hearts with reoxygenation, but recovered slightly in hearts previously exposed to chronic hypoxia (9% relative to end‐hypoxia, *P* = 0.039) or DCA treatment (8% relative to end‐hypoxia, *P* = 0.001). Cardiac glycogen content was no different following chronic hypoxia or DCA treatment compared to normoxic controls. Acute hypoxia resulted in a 41% fall in cardiac glycogen content in normoxic animals (Fig. 5 *D*, *P* = 0.002), with no significant effect of chronic hypoxia, but DCA spared cardiac glycogen (*P* = 0.007) compared to hearts from normoxic animals. This difference was maintained during reoxygenation (*P* = 0.022 *vs*. normoxic controls), but also compared to hearts from vehicle‐fed chronically hypoxic animals (*P* = 0.007). Cardiac muscle lactate content was not altered with either chronic hypoxia or chronic hypoxia with DCA treatment (Fig. 5 *E*). However, at the end of acute hypoxia lactate content was 20% less in hearts from chronically hypoxic mice compared to normoxic controls (*P* = 0.038), and 47% less in hearts from chronically hypoxic, DCA‐fed animals (*P* = 0.001). These responses were not altered with reoxygenation. Total creatine was unaltered with chronic hypoxia or DCA treatment (Fig. 5 *F*), but DCA treatment in chronic hypoxia preserved total creatine during acute hypoxia (45% greater than normoxic control or chronic hypoxia groups, *P* = 0.001) and following reoxygenation (40% greater than normoxic control or chronic hypoxia groups, *P* = 0.002).

## Discussion

This study aimed to identify the metabolic physiology of hypoxic cardiac tolerance in response to the pharmacological activation of PDC and its flux with dichloroacetate. Information on the role of the PDC in cardiac adaptation to chronic hypoxia is scarce. It is unclear how the chronically hypoxic heart responds functionally and metabolically to acute stress following hypoxic adaptation, with a variety of results reported in the literature, and the role of the PDC is undetermined. In an acute hypoxic challenge, DCA‐mediated PDC activation did not improve function, probably due to the fact that a large increase in PDC activation was not matched by a similar increase in flux through the PDC reaction. However, in the chronically hypoxic heart, where the tolerance to acute hypoxia was substantially reduced, simultaneous DCA‐mediated activation of the PDC with chronic hypoxia stockpiled acetylcarnitine, a marker of elevated PDC flux. When subsequently exposed to acute hypoxia, this acetylcarnitine surplus was exhausted. The use of these acetyl groups resulted in improved function both during acute hypoxia and during reoxygenation.

### Acute hypoxia

We first assessed how activation of the PDC via DCA infusion affected the cardiac response to acute hypoxia. Acute hypoxia resulted in considerable cardiac damage, as determined by the impaired recovery during reoxygenation. Despite activating the PDC 4‐fold with an infusion of DCA during acute hypoxia, limited evidence of flux was apparent, with only a small increase in acetylcarnitine content. This did not improve cardiac function, which was also true of DCA infusion during reoxygenation alone – acetylcarnitine accumulation was also limited in this condition. These findings contrast with the positive effects of DCA infusion reported in global ischaemia experiments, where recovery was improved with DCA infusion at the onset of reperfusion (McVeigh & Lopaschuk, 1990; Randall *et al*. 1998; Wambolt *et al*. 2000). One difference between these ischaemic protocols and the acute hypoxic protocol used in our studies is that contractile function was sustained throughout hypoxia, albeit at 20% of baseline function, in contrast to ischaemic studies, where function is typically reported as negligible (Schroeder *et al*. 2009), even when some coronary flow was maintained (Sidell *et al*. 2002). During hypoxia, this function was supported by significant fatty acid oxidation, indicating that a significant portion of ATP production was via oxidative phosphorylation, and it may be that a change in redox status subsequently affected recovery from acute hypoxia. The infusion of DCA during reoxygenation suppressed fatty acid oxidation – the mechanism for this may be via reduced free carnitine content due to acetylation of the carnitine pool (Stephens *et al*. 2007), or increased malonyl‐CoA levels (Saddik *et al*. 1993). As recovery of function was unaltered, cardiac energy supply was presumably compensated for by increased carbohydrate metabolism.

### Chronic hypoxia

Chronically hypoxic hearts were functionally less tolerant of subsequent acute hypoxia, during the acute hypoxic period, and exacerbated by reperfusion. Reduced hypoxic tolerance of the intact beating rat heart following 2 weeks of housing in 10% F iC O2 has been reported previously (Milano *et al*. 2002), although the mechanisms underlying such impairment were not investigated. In contrast, others report improved tolerance following hypoxic exposure in primary cardiomyocytes exposed for 48 h (Silverman *et al*. 1997), or in papillary muscle exposed to hypoxia for up to 74 weeks (La Padula & Costa, 2005), with improvements observed after 1 week. The cause of the increased damage seen in our experiments does not appear to be via alterations in substrate metabolism, although we found an increased reliance on glycolysis in reoxygenation, consistent with metabolic inflexibility following chronic hypoxia (Cole *et al*. 2016).

### Chronic hypoxia does not result in reduced PDC function

We hypothesised that sustained *in vivo* hypoxia would result in a fall in PDC activity and flux, thus limiting mitochondrial respiration and attenuating reactive oxygen species generation (Kim *et al*. 2006; Papandreou *et al*. 2006). However, we did not observe any modification of PDC activity or flux following 21 days of hypoxia. Le Moine *et al*. (2011) showed that PDC activity of murine skeletal muscle fell following 24 h of exposure to the equivalent of 13% $F_{iO_{2}}$, but had recovered with 7 days of sustained hypoxic exposure. This mechanism is possibly due to the transient nature of the oxygen sensing transcription factor, hypoxia‐inducible factor 1α stabilisation, also shown by Le Moine *et al*. (2011), previously reported by Stroka *et al*. (2001) in brain, liver and kidney, and demonstrated in beating cardiac cells by Ambrose *et al*. (2014). Hence it would appear impairment of PDC function is part of the early cellular response to hypoxia, with PDC function returning to normal with sustained restriction of oxygen.

### The role of acetylcarnitine in hypoxic tolerance

Treatment with DCA during chronic hypoxia improved cardiac function, both during acute hypoxia and in reoxygenation. DCA treatment has been seen to have beneficial effects on systolic function following ventricular fibrillation (Azam *et al*. 2015) and diastolic function in the diabetic heart (Le Page *et al*. 2015), where PDC flux is impaired. In our study, chronic treatment of the hypoxic heart with DCA resulted in evidence of increased flux through the PDC reaction, with a doubling of cardiac acetylcarnitine content. In situations where acetyl‐CoA production exceeds utilisation in the Krebs cycle, acetylcarnitine accumulates in the cytosol via the actions of carnitine acetyl transferase (CAT) (Constantin‐Teodosiu *et al*. 1991a), thereby acting as an acetyl group buffer. Schroeder *et al*. (2012) found that around half of all cardiac acetyl‐CoA produced via pyruvate is cycled through CAT before being committed to the TCA cycle, supporting the idea that the CAT system is doing more that acting as a disposal route for excess acetyl‐CoA.

It has been shown previously that increasing the acetylcarnitine pool prior to the onset of contraction under ischaemic conditions can improve skeletal muscle fatigue resistance (Timmons *et al*. 1997; Roberts *et al*. 2005) by compensating for an acetyl group deficit at the onset of muscle contraction that exists as a result of inertia in activation of the PDC (Greenhaff *et al*. 2002). Loss of CAT function also prevents improvements in skeletal muscle performance with increased acetyl group availability (Seiler *et al*. 2015). With sustained contraction, acetylcarnitine content increases (Roberts *et al*. 2005), as mitochondrial acetyl‐CoA levels are increased via irreversible oxidative decarboxylation of pyruvate via the PDC and increased oxidation of fatty acids. With prolonged exercise and the development of limitation in substrate supply, skeletal muscle acetylcarnitine levels fall (Watt *et al*. 2002). In our cardiac study, acetylcarnitine fell during acute hypoxia, possibly as a result of allosteric inhibition of PDC flux via a change in the redox state, and the subsequent use of acetylcarnitine as a supply of acetyl‐CoA to support a limited amount of substrate oxidation. The mismatch between activation of PDC, and PDC flux, has been previously shown to occur in ischaemic skeletal muscle, where the inability to reoxidise NADH was shown to have no effect on the activity of the PDC, but inhibited its flux and hence accumulation of acetyl groups (Constantin‐Teodosiu *et al*. 1993).

Our finding that increasing acetyl group availability improves cardiac function supports the hypothesis that acetylcarnitine buffers acetyl‐CoA levels (Constantin‐Teodosiu *et al*. 1991a; Seiler *et al*. 2015). This hypothesis is based primarily on work on skeletal muscle, and building on previous work on cardiac ischaemia (McVeigh & Lopaschuk, 1990; Greenhaff *et al*. 2004), we believe our study to be the first to provide evidence that increasing acetyl group availability improves cardiac function during and following hypoxia. The stockpile of cardiac acetylcarnitine we observed following prolonged DCA administration concurrent with chronic hypoxia was exhausted by the end of 30 min of acute hypoxia. The significant oxidative phosphorylation evident throughout this acute hypoxic period suggests that this acetylcarnitine was being used to maintain acetyl‐CoA for use in the Krebs cycle, thus sparing glycogen and maintaining ATP, maintaining cardiac function during hypoxia and reducing hypoxic damage.

### Summary

We found in this study that chronic hypoxia exacerbated the damage from subsequent acute hypoxia, but that this was counteracted by activation of PDC during chronic hypoxic exposure. This improved cardiac performance was associated with stockpiling of PDC‐mediated acetylcarnitine, which was depleted during acute hypoxia, and reduced hypoxic damage. This finding suggests that activation of PDC may have therapeutic potential in cardiac disease with a hypoxic component.

## Additional information

### Competing interests

None declared.

### Author contributions

M.K.H. contributed to the acquisition, analysis and interpretation of data, and drafted versions of the final manuscript. D.C.‐T. analysed and interpreted data and revised the manuscript critically for important intellectual content. P.L.G. contributed to both the design of the work and revising the manuscript critically for important intellectual content. M.A.C. conceived the study, contributed to its design, interpreted data and drafted the work. All experimental work was conducted in the Medical School of the University of Notttingham, UK. All authors approved the final version of the manuscript and agree to be accountable for all aspects of the work in ensuring that questions related to the accuracy or integrity of any part of the work are appropriately investigated and resolved. All persons designated as authors qualify for authorship, and all those who qualify for authorship are listed.

### Funding

This study was supported by the University of Nottingham and a Biotechnology and Biological Sciences Research Council (BBSRC) award BB/F016956/1.
